# Deep brain stimulation (DBS) as a therapeutic approach in gait disorders: What does it bring to the table?

**DOI:** 10.1016/j.ibneur.2023.05.008

**Published:** 2023-05-19

**Authors:** Ramtin Pourahmad, Kiarash Saleki, Mohammadreza Esmaili, Arian Abdollahi, Parsa Alijanizadeh, Mehrad Zare Gholinejad, Mohammad Banazadeh, Mona Ahmadi

**Affiliations:** a School of Medicine, Tehran University of Medical Sciences, Tehran, Iran; b Network of Immunity in Infection, Malignancy and Autoimmunity (NIIMA), Universal Scientific Education and Research Network (USERN), Tehran, Iran; c Student Research Committee, Babol University of Medical Sciences, Babol, Iran; d Department of e-Learning, Virtual School of Medical Education and Management, Shahid Beheshti University of Medical Sciences(SBMU), Tehran, Iran; e USERN Office, Babol University of Medical Sciences, Babol, Iran; f Pharmaceutical Sciences and Cosmetic Products Research Center, Kerman University of Medical Sciences, Kerman, Iran; g Department of Neurology, School of Medicine, Kurdistan University of Medical Sciences, Sanandaj, Iran

**Keywords:** Deep brain stimulation, Gait disorder, Gait imbalance, Neurosciences, Neurodegeneration

## Abstract

Gait deficits are found in various degenerative central nervous system conditions, and are particularly a hallmark of Parkinson’s disease (PD). While there is no cure for such neurodegenerative disorders, Levodopa is considered as the standard medication in PD patients. Often times, the therapy of severe PD consists of deep brain stimulation (DBS) of the subthalamic nucleus. Earlier research exploring the effect of gait have reported contradictory results or insufficient efficacy. A change in gait includes various parameters, such as step length, cadence, Double-stance phase duration which may be positively affected by DBS. DBS could also be effective in correcting the levodopa-induced postural sway abnormalities. Moreover, during normal walking, interaction among the subthalamic nucleus and cortex —essential regions which exert a role in locomotion— are coupled. However, during the freezing of gait, the activity is desynchronized. The mechanisms underlying DBS-induced neurobehavioral improvements in such scenarios requires further study. The present review discusses DBS in the context of gait, the benefits associated with DBS compared to standard pharmacotherapy options, and provides insights into future research.

## Highlights

• Deep brain stimulation (DBS) has been established as an efficacious therapy for Parkinson's disease (PD).
• Gait disturbance is implicated in many nervous system disorders.
• DBS can enhance the results obtained via standard therapeutic regimen.
• The efficacy of DBS should be evaluated, as progression of neurodegeneration limits DBS benefits.

## 1 Introduction

The act and style of walking, known as gait, is a learned complicated motor skill that aids mobility. Gait needs the integration of locomotion mechanisms with those of balance, motor control, cognition, and musculoskeletal function, even though it can be done automatically and without conscious effort (Nutt, 2013). Gait abnormalities, which are frequently coupled with postural instability, are a significant disability and source of suffering for patients. The elderly are more prone to falls because their reserves for balance and stride are depleted (Tinetti, 2003). Gait abnormalities are widespread among the elderly, and their frequency rises with age. The prevalence of normal gait decreases from 85% at the age of 60, to 18% by the age of 85 (Bloem et al., 1992, Sudarsky, 2001). Gait problems can be life-threatening. The most well-known consequence is falling, which is frequently triggered by an underlying gait issue. Accidental falls can result in anything from minor bruising to serious fractures or head injuries. Reduced mobility, which leads to a loss of independence, is another key consequence. This immobility is frequently exacerbated by a fear of falling, further immobilizing patients and negatively impacting their quality of life (Jørstad et al., 2005). It is intriguing that gait is not limited to non-treatable neurodegenerative disorders. The CNS involvement experience of COVID-19 pandemic, for which a definitive pharmaceutical treatment is also lacking, corroborates this fact (Klein et al., 2020, Saleki et al., 2020, Rostamtabar et al., 2021, Rahmani et al., 2020, Li et al., 2021, Mohseni Afshar et al., 2022, Saleki et al., 2021, Saleki et al., 2022b). Deep brain stimulation (DBS) has been established as an efficacious therapeutic approach for Parkinson's disease (PD), essential tremor (ET), and dystonia. Obsessive-compulsive disorder (OCD) and medically refractory epilepsy are two conditions for which it has recently been approved (Cooper, 1955, Lachenmayer et al., 2019). First use of what is now known as contemporary DBS was in 1980, when electrical stimulation of the midbrain and basal ganglia was used to control intention tremor (Brice and McLellan, 1980). The introduction of DBS has sparked a resurgence in functional neurosurgery. More than 140,000 patients are thought to have received DBS, with more than 12,000 additional patients being treated per year throughout the world (Lee et al., 2019).

## 2 Deep brain stimulation

### 2.1 Definition

The insertion of electrodes into deep brain areas to manipulate neural function in order to treat neurological disorders is referred to as DBS. These electrodes are electrically connected to an implanted pulse generator (IPG), normally hidden beneath the clavicle. An IPG has a battery and electronic components that produce electrical stimulation and can be adjusted by patients or clinicians (Pycroft et al., 2018). An MRI scan or CT scan affirms the electrode's position after its stereotactic placement into the specific brain region. The most significant component in the procedure's effectiveness is proper electrode placement (Bötzel and Kraft, 2010).

The origins of DBS can be traced back to as early as 1900 s, when breakthroughs in animal cerebral cortex stimulation set the groundwork for cortical functional localization as we know it today. At that time, the first stereotactic case was established, allowing researchers to experiment with stimulation of deeper brain regions (Schwalb and Hamani, 2008). The first report that demonstrated the effect of the subthalamic nucleus (STN) lesioning in alleviating motor symptoms was a study done on a monkey with PD (Schwalb and Hamani, 2008). Among the first applications of thalamic DBS was a case for treating tremor with utilizing a fully implantable neurostimulation system which was a technological requirement for a quick switch to alternative targets and indications (Benabid et al., 1987, Pollak et al., 1993, Siegfried and Lippitz, 1994).

Different mechanisms for DBS have been proposed in our review of literature. Neural inhibitory response is one of the mechanisms of effect in DBS that several reports have demonstrated a reduction in neuronal activity nearby the DBS site (Vitek, 2002, Mayberg et al., 2005). However, other studies have shown that DBS can sometimes even accentuate neural activity, which contradicts these findings (McIntyre et al., 2004). Informational lesion is another candidate for explaining the mechanism of DBS, the irregular and fluctuating nature of normal brain activity facilitates the transmit of information, and DBS converts this irregular activity to a regularized, less-variant one (Grill et al., 2004), decreasing the number of data exchanged between network nodes (Dorval et al., 2008). This might make the overall network function better. An alternative explanation of the mechanism of action of DBS is the theory of disruption of neuronal transmission between different parts of the brain. In the cases of PD, multiple investigations have found oscillatory activity and aberrant bursts in Globus pallidus (GPi) neurons (Bilge et al., 2018). The transportation of these abnormal signals from the GPi to the thalamus and then to the motor cortex can be concluded to be the cause of PD motor symptoms. As a result, if the transmission of these abnormal signs is disrupted, the motor symptoms will likely resolve (Stefani et al., 2019). In this regard a previous study investigated the behavior of GPi neurons during GPi-DBS in response to cortical stimulation (Chiken and Nambu, 2013). This study showed that GPi-DBS leads to a decrease in cortical responses, which suggests that GPi-DBS blocks the flow of information through the GPi. This disruption of neural transmission has been linked to relief of motor symptoms.

DBS has significant advantages over other neuromodulation surgical techniques as a therapeutic tool. The non-lesional character of DBS, the ability to adjust stimulation parameters to maximize benefit while minimizing detrimental effects, and the ability to directly engage with the circuit pathophysiology that produces the symptoms are just a few of the benefits of DBS. Despite its benefits, DBS is still an invasive surgical procedure with minimal but potentially substantial complications, like cerebral bleeding and infection (Lozano et al., 2019). DBS is an effective treatment strategy for a vast variety of neurological disorders, such as PD (Obeso et al., 2001, Rodriguez-Oroz et al., 2005), essential tremor (Koller et al., 2001, Benabid et al., 1991), and dystonia (Kupsch et al., 2006, Vidailhet et al., 2005) that became the standard of care for mentioned conditions after receiving Food and Drug Administration (FDA) and European conformite (CE) approval (Cagnan et al., 2019). Chronic pain was the first indication for chronic DBS, long before it was commonly used to treat movement disabilities (Levy et al., 2010). Aside from movement disorders, the clinical use of DBS has been practiced in several neuropsychiatric disorders. For instance, in Tourette’s syndrome, DBS has been shown to minimize or eliminate behavioral symptoms (Bajwa et al., 2007, Visser-Vandewalle et al., 2003, Kuhn et al., 2007). Nevertheless, more studies with randomized controlled designs are needed for Tourette's syndrome. Major depression (Schlaepfer et al., 2008), OCD (Fontaine et al., 2004), and epilepsy (Davis and Emmonds, 1992, Velasco et al., 1989) are among the other neuropsychiatric conditions that have been suggested to be a candidate for the use of DBS.

Depending on the condition that we are tackling, different brain regions are targeted during DBS (Williams and Okun, 2013). Some of these DBS targets are the standard use while others are merely experimental. STN as the most common site for DBS is a well stablished target for PD (Obeso et al., 2001, Limousin et al., 1998). According to previous researches in this regard, another candidate that may require STN-DBS is OCD (Rappel et al., 2018). GPi as an another brain target in DBS implantation investigated in PD (Volkmann et al., 2009), Dystonia (Kupsch et al., 2006), and Tourette syndrome (Neumann et al., 2018). Another accepted sites for stimulation are ventrolateral thalamus (VL) in essential tremor (Benabid et al., 1991, Rehncrona et al., 2003) and PD. Targeting zona incerta,although not widely accepted in clinic, has been showing promising results in patients with PD (Ossowska, 2020) and essential tremor (Philipson et al., 2019, Eisinger et al., 2018).

## 3 DBS as a therapeutic approach in gait disorders

Walking is a multidimensional activity that is directed through both voluntary and automatic mechanisms that are influenced by emotions. Acute development of a gait disturbance can imply a cerebrovascular or other acute nervous system impairment, as well as systemic disorders or pharmaceutical side effects (Alexander and Goldberg, 2005). The most prevalent neurological reasons comprise sensory ataxia due to polyneuropathy, parkinsonism, and frontal gait problems related to subcortical vascular encephalopathy or diseases connected with memory loss (Alexander and Goldberg, 2005, Snijders et al., 2007). Its neural control is thus based on a variety of circuits, with the basal ganglia and cerebellum playing critical roles (Takakusaki et al., 2008). All the structures and activities necessary for proper gait, including as locomotor function for initiation and maintaining the cadence of gait, stability, postural reflexes, sensory function and sensorimotor connection, motor function, the musculoskeletal system, and cardiorespiratory processes, must be intact (Jankovic and Tolosa, 2003, Takakusaki et al., 2004).

**Fig. 1:**
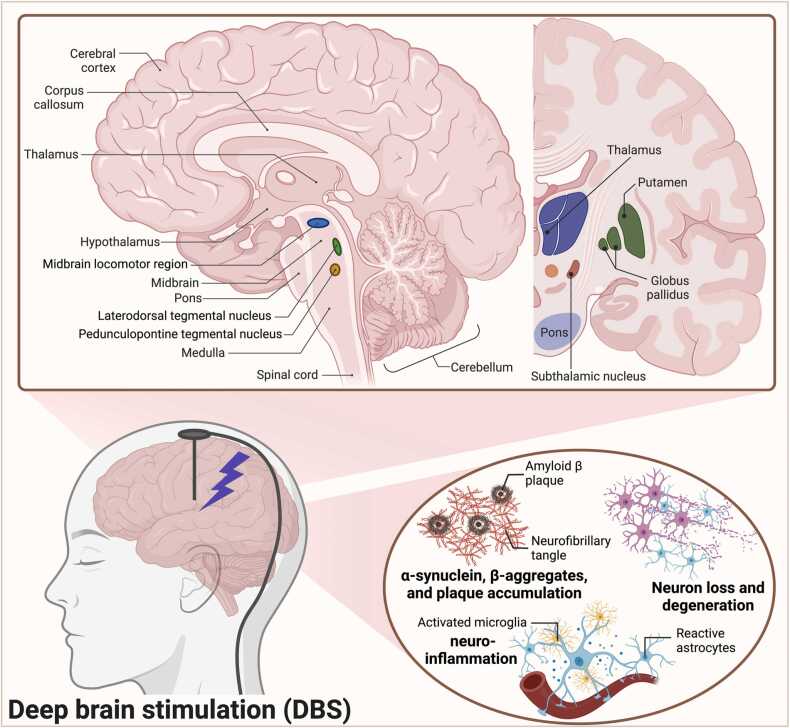
Deep brain stimulation and brain structure. This figure shows the brain structure involved in Parkinson's disease, like globus pallidus, midbrain locomotor region, Laterodorsal tegmental nucleus, pedunculopontine tegmental nucleus, and subthalamic nucleus. Deep brain stimulation could stimulate these parts to improve symptoms of Parkinson's disease. Moreover, this figure shows neuroinflammation, neuron degeneration, and accumulation of α-synuclein and β-aggregates are histopathological features of Parkinson's disease. Created with BioRender.com.

The mesopontine tegmentum (MPT) is a cellular region located at the intersection of the mesencephalon and the pons. The pedunculopontine tegmental nucleus (PPN) and the laterodorsal tegmental nucleus (LTN) are two cholinergic nuclei in the mesopontine tegmentum (Maskos, 2008). Physiologically critical parts engaged in posture and gait control can be found in the lateral section of the mesopontine tegmentum (Takakusaki et al., 2003). Two functionally recognized regions in the mesopontine tegmentum are involved in the modulation of locomotion and postural muscle tone. The MLR (midbrain locomotor region) that relates substantially to the cuneiform nucleus and in proximity to the dorsal side of the pedunculopontine pontine tegmental nucleus and the muscle tone inhibitory region in the ventrolateral section of the PPN as the other part. MLR stimulation elicited membrane depolarization demonstrating that locomotion involves the involvement of both the locomotor as a rhythm generating system and the muscle tone as an excitatory system (Takakusaki et al., 2004, Takakusaki et al., 2006). We show the brain structure involved in PD and histopathological features of PD (Fig. 1).

In basal ganglia dysfunction such as Parkinson’s disease, gait deficiency is defined by latency in gait commencement, narrow steps, propulsions, and poor balance when walking (Murray et al., 1978, Pahapill and Lozano, 2000). Gait and balance issues have identified as significant therapeutic considerations in people with PD (Obeso et al., 2001). Dopaminergic medications or DBS can be used to manage the dopamine-responsive features of gait impairment in the primary phases of the disease. DBS in several basal ganglia target nuclei has been well confirmed as a therapeutic approach for PD motor symptoms and consequences (Follett et al., 2010). It has been shown that excitation of the STN or GPi can relieve motor clinical signs and is even more successful than the best medicine in enhancing the quality of life of individuals with severe PD. Tremor, stiffness, and limb bradykinesia have emerged as the most important treatment issues in end stage PD, as they react poorly to pharmacologic therapy or surgery, restrict movement, and have a significant effect on quality of life (Grabli et al., 2013). More than half of the patients will have symptoms that are resistant to medicine and will develop drug-induced dyskinesia along the phase of the disease. A major meta-analysis demonstrated that, while patients who underwent STN-DBS had better motor symptoms than those who received GPi-DBS, the results was not statistically significant (Weaver et al., 2005). The Unified Parkinson's Disease Rating Scale (UPDRS) reported significant decline in postural instability and gait disorder symptoms in the first findings of STN-DBS for PD patients (Goetz et al., 2008). The considerable improvement in stride length generated by STN-DBS in the medication off condition, which was leading overall elevation in gait velocity, was a common result in most investigations, but cadence remained mainly intact (Maskos, 2008). STN-DBS enhanced trunk and leg vertical arrangement and induced a bigger backward and lateral dislocation of the center of pressure, indicating a better physiological expression of the underlying muscle synergies either during or before gait initiation (Crenna et al., 2006). Within the first year following STN-DBS implantation, more than half of the individuals with PD showed an improvement in their gait and less postural instability (Limousin et al., 1998). Following the long-term deterioration of stimulation-resistant gait abnormalities and freezing following STN-DBS therapy, the effects of energy content and frequency adjustment investigated. Results showed that high voltage stimulation (60 Hz) prompt much higher gait improvement than lower voltage stimulation (130 Hz) (Moreau et al., 2008). Switching to 60 Hz low frequency voltage eased freezing of gait significantly (Xie et al., 2012).

Latest evidence on the cerebellum's function in PD and its direct disynaptic connections to the basal ganglia attracted attentions to the VIM as a significant cerebellar information relay (Wu and Hallett, 2013). VIM stimulation has been offered as an alternative for thalamotomy, with the primary goal of suppressing tremor and only a slight improvement in gait and axial symptoms (Ferraye et al., 2008). According to previous trials, VIM reduced tremor in around 85% of cases; although, akinesia and rigidity were only marginally alleviated, and clinical axial symptoms, such as gait scores, remained unaltered (Limousin et al., 1999).

Previous systematic reviews and meta-analyses evaluating the eﬀectiveness of GPi-DBS and STN-DBS on PD patients have demonstrated that no significant differences in therapeutic performance on PD motor symptoms between the GPi-DBS and the STN-DBS have been found (Follett et al., 2010, Weaver et al., 2005, Peng et al., 2018). Meanwhile, another study implicated that, while the STN-DBS may be superior to the GPi-DBS in enhancing motor function and daily living activities for PD patients when they are not on medication, the inverse is true when they are on medication (Xu et al., 2016).

With growing understanding that it may be implicated in the development of several motor diseases, the PPN has sparked a lot of study in the recent decade (Jenkinson et al., 2009). PPN-DBS as another therapeutic approach in PD patient to relieve their gait disabilities and enhancement of motor axial symptoms was first utilized in 2007. Concurrent to the insertion of STN electrodes, the first implantation of bilateral electrodes in both PPN was done in six PD cases. Results showed an overall 32% improvement in UPDRS score in off medication state during 3–6 months after implantation (Stefani et al., 2007). In another previous trial, no improvement in a group of 6 PD patients who underwent unilateral PPN-DBS was detected; however, patients reported fewer falls and freezes (Moro et al., 2010). Additionally, a recent study suggests that low-frequency stimulation of the substantia nigra reticulata in combination with DBS in the subthalamic nucleus could be an effective treatment for freezing during the "on" period in PD (Valldeoriola et al., 2019).

## 4 Comparing the effect of STN-DBS and Levodopa

The dopamine (DA) precursor molecule L-dopa is known as the gold standard therapy for PD from the late 1960’s when Cotzias et al. described L-dopa ’s efficiency for symptomatic management of PD. From then, L-dopa treatment has improved both the life expectancy and quality of life of PD patients. L-dopa could pass the Blood-Brain-Barrier (BBB) and is transformed to DA in the Central Nervous System (CNS) in Dopa Decarboxylase (DDC) expressing neurons. In spites of the fact that multiple DA substitution treatments are now accessible, L-dopa is considered the most efficient medicine for controlling motor signs in PD. Yet, L-dopa treatment has some caveats. As the disease develops long-term therapy with L-dopa is related to motor difficulties, i.e., motor fluctuation (the therapeutic effect duration once each dose diminishes) and dyskinesia (involuntary movements). These motor difficulties are observed in most of PD patients, who are being treated with L-dopa for longer than five years. Furthermore, probable neuroprotective versus neurotoxic impacts of L-dopa are being debated for years (Bogetofte et al., 2020).

Considering more than 140,000 patients treated around the globe, STN-DBS is a recognized therapy for motor difficulties in PD. Decline in levodopa equivalent daily dose (LEDD) and other dopaminergic drugs is observed profoundly after STN-DBS and is now considered an “anticipated benefit” of such surgical modality. This theory is based on the logic that STN-DBS could decrease PD principal signs to a comparable amount than L-dopa and that decrement of drugs ameliorates postoperative dyskinesia. By contrast, drug dose reduction could bring about other difficulties, such as apathy and depression, which induces doubt about forceful reduction of dopaminergic treatments in patients treated with STN-DBS. Taken together, there is a challenging debate regarding STN-DBS in PD (Vizcarra et al., 2019).

Even though, the efficiency of DBS on segmental motor symptoms, i.e*.*, appendicular akinesia, tremor, and rigidity is well described, its impact on axial disability is still debated. Some authors demonstrated an enhancement of gait, posture, and balance control subsequent to STN-DBS, or GPi (with a bolder enhancement with STN-DBS). While these signs were sensitive to L-dopa therapy prior to surgery (Klein et al., 2020, Saleki et al., 2020, Rostamtabar et al., 2021, Rahmani et al., 2020, Li et al., 2021, Mohseni Afshar et al., 2022, Saleki et al., 2021, Saleki et al., 2022b, Cooper, 1955, Lachenmayer et al., 2019, Brice and McLellan, 1980, Lee et al., 2019, Pycroft et al., 2018, Bötzel and Kraft, 2010, Schwalb and Hamani, 2008), those effects tend to taper-off along time. Additionally, several authors propose that STN-DBS may cause or worsen postural instability with falls and freezing of gait in PD patients. A different probability, indicated by personal experiences and several studies in the literature, is that in a subsection of patients the concomitant effect of L-dopa and STN-DBS could exacerbate gait by inducing “on” lower body dyskinesia. Even though the additive/synergic impact of L-dopa and STN-DBS on gait patterns might remain debated in the literature, in medical practice it is clearly evident, especially with an increase in dyskinesia either from L-dopa, stimulation, or both. The lesioning effect is comparable to the concomitant L-dopa and stimulation effect, at situations that require a rapid reduction of L-dopa amount in the immediate postoperative phase to minimize acute dyskinesia. Most patients diagnosed with progressed PD exposed to DBS are fairly prone to dyskinesia, with a subgroup within them demonstrating lower extremities predominant dyskinesia. Lower limbs higher involvement might have also been related with the topography (or otherwise theorized as the “homunculus of the STN”) by which, based on the electrode position, some regions within the STN receive differing stimulus. Through experimentation, researchers observed in a specific set of patients that using stimulation along with L-dopa resulted in dyskinesia in the lower extremities. The symptoms of dyskinesia manifested as walking-evoked hyperkinetic lower extremity movement (e.g., a strange acting instantaneous hip and knee flexion), walking-induced one-sided foot eversion, along with “ON-freezing” of gait. This observation is backed by several patients whose freezing of gait vanished due to complete discontinuation of L-dopa therapy and stayed treated exclusively with stimulation. Overall, the effect of STN-DBS versus L-dopa shows a paradoxical trend that relies on many factors (Cossu and Pau, 2017).

## 5 Insight for the future

Vast majority of researches in literature regarding the effect of DBS on gait disorders was focused on the STN-DBS and GPi-DBS, having said that there is a need to thoroughly investigate and distinguish the reasons behind the ineffectiveness of STN-DBS in treating gait problems specifically determining that the failure in treating postural instability and gait disorder is due to deterioration of the patient’s illness or the fact that the therapy was incapable of improving gait symptoms. Also more randomized, controlled trials with extended follow-up are required to explore the effect of GPi-DBS and STN-DBS on PIGD (postural instability and gait difficulty), as well as developing guidelines and preoperative evaluations to determine the most suitable PD patients for the various surgical procedures.

Aside from STN and GPi there are other brain regions that are targeted in DBS surgery for gait problems, including PPN, centro-median thalamic nucleus, and the zona incerta (Piper et al., 2005, Allert et al., 2001, Mazzone et al., 2014). There are few studies that have investigated the effectiveness of DBS on the zona incerta and PPN as designated spots in PD, especially PPN as its role in neural circuits could be an intriguing option for people with PD experiencing subtle cognitive abnormalities or undergone failed attempts of STN-DBS. Thus, future effort must be made on assessing the impact of stimulation on these specific spots using accurate and valid gait performance indicators. Understanding the difference among targets (GPi, STN, PPN, etc.) will require deeper investigation into their impact on gait symptoms and will assist to choose the best target location for stimulation. There are some early but encouraging findings that are recently issued regarding the effectiveness of coupled SNr-DBS and STN-DBS (Weiss et al., 2013, Weiss et al., 2011). These studies demonstrated that the coupled stimulation method in question was safe with no meaningful side effects (Weiss et al., 2013). The advantages of combining STN-DBS and SNr-DBS will have to be studied in larger populations, as they provide alternative therapeutic solution for individuals with STN-DBS experiencing severe gait deficits. We recommend further research on specific cells in the CNS and their relation with DBS. DBS leads to moderate local progenitor cellular proliferation and can affect the overall number of induced microglia. This may be of clinical importance in PD cases, as it is believed to progress by neuroinflammatory mechanisms associated with microglia, cytokines, and the complement system. More research is needed to decipher the cellular behavior of microglia in various activation conditions and their potential to mediate neurogenesis in normal and disease states (Javanmehr et al., 2022, Vedam-Mai et al., 2016). While our discussion focuses on new methods, herbals have been investigated in a wide range of studies (Amir et al., 2007, Amirghofran et al., 2007, Azadmehr et al., 2013a, Azadmehr et al., 2013b, Azadmehr et al., 2014, Latifi et al., 2022). Traditional approaches also deserve more research as add-on therapy in PD. We suggest a combinational approach utilizing traditional medicine, conventional therapies, and new treatments like DBS (Srivastav et al., 2017, Li et al., 2013, Chen et al., 2007).

A multifaceted targeting of gait in PD or other important conditions could provide optimal results, it may be beneficial to utilize inflammation modulating pharmaceutical and natural therapies, targeting inflammasomes, toll-like receptors (TLRs) and adaptive immunity, and even opioid brain receptors (Saleki et al., 2022b, Latifi et al., 2022, Chen et al., 2019, Rasoulinejad et al., 2020, Karkhah et al., 2021, Huang et al., 2018, Ranjbar et al., 2021, Saleki et al., 2022b, Gale and Martyn, 2003). Reduction of environmental stress could also serve as a reasonable adjunct prescription for such patients (Fukui et al., 2018, Razavinasab et al., 2022). These immune elements modulate hyperinflammation and significantly contribute to neurological disorders. Immunoinformatics could help to develop immune-mediating therapeutics to this aim rapidly against CNS disorders (Saleki et al., 2022a, Rahmani et al., 2021, Saleki et al., 2022c, Saleki et al., 2022d).

## 6 Conclusion

Gait disturbance is implicated in many nervous system disorders. DBS can enhance the results obtained via standard therapeutic regimen. Also, the efficacy of DBS should be evaluated, considering that as a neurodegenerative condition progresses the nerve cells may be lost, limiting the benefits associated with DBS. The neurodegenerative processes are multifaceted. For instance, even one complication such as gait disturbance results from damage to many brain regions. Ultimately, the clues provided by the rapidly increasing evidence warrants future research utilizing DBS in conjunction with standard treatments that may be symptom relievers or neurodegeneration-slowing for optimal results.

## Ethics approval

Not applicable.

## CRediT authorship contribution statement

Ramtin Pourahmad, Kiarash Saleki, Mohammadreza Esmaili, Arian Abdollahi, Mehrad Zare Gholinejad, and Mohammad Banazadeh drafted the manuscript. Kiarash Saleki and Parsa Alijanizadeh made the figure. Kiarash Saleki prepared the final draft and critically appraised the manuscript. Mona Ahmadi supervised the project, drafted the manuscript, and critically appraised the manuscript. Figure created with BioRender.com.

## Consent to participate

Not applicable.

## Funding

None.

## Declaration of interests

The authors report there are no competing interests to declare.
